# Suppression of Airway Allergic Reactions by a Photocatalytic Filter Using Mouse Model

**DOI:** 10.3390/toxics10010040

**Published:** 2022-01-15

**Authors:** Taisuke Tomonaga, Hiroto Izumi, Chinatsu Nishida, Kaori Kato, Kazuhiro Yatera, Etsushi Kuroda, Yasuo Morimoto

**Affiliations:** 1 Institute of Industrial Ecological Sciences, University of Occupational and Environmental Health, 1-1 Iseigaoka, Yahata-nishi-ku, Kitakyushu 807-8555, Japan; h-izumi@med.uoeh-u.ac.jp (H.I.); yasuom@med.uoeh-u.ac.jp (Y.M.); 2 Department of Respiratory Medicine, University of Occupational and Environmental Health, 1-1 Iseigaoka, Yahata-nishi-ku, Kitakyushu 807-8555, Japan; c-nishi@med.uoeh-u.ac.jp (C.N.); kato-kr@med.uoeh-u.ac.jp (K.K.); yatera@med.uoeh-u.ac.jp (K.Y.); 3 Department of Immunology, Hyogo College of Medicine, 1-1 Mukogawacho, Nishinomiya 663-8131, Japan; kuroetu@hyo-med.ac.jp

**Keywords:** photocatalyst, ovalbumin, allergic reactions, high-velocity oxygen fuel spraying, TiO_2_, intratracheal instillation, mice

## Abstract

Photocatalytic filters installed in air purifiers have been used to purify spaces by decomposing allergenic substances. However, we have not found any reports that evaluate the effectiveness of photocatalytic filters in suppressing allergic reactions in living organisms. In this study, we intratracheally instilled ovalbumin (OVA) into OVA-sensitized mice after the OVA was photocatalyzed by a titanium dioxide (TiO_2_) filter, and verified the experimental model for evaluating the allergy-suppressing effect of photocatalysts. Mice were sensitized to OVA (10 µg/mouse) four times, and were intratracheally instilled with OVA (10 µg/mouse) after photocatalysis three times. Non-sensitized animals were instilled with normal saline following the same exposure schedule. The mice were dissected 24 h after final exposure. The OVA after photocatalysis significantly decreased the number of eosinophils in bronchoalveolar lavage fluid, and the concentration of OVA-specific IgE and IgG1 in serum, which were elevated in untreated OVA. Moreover, our experimental model showed the suppression of allergic reactions in mice, along with the decomposition of OVA after photocatalysis using the photocatalytic filter. Taken together, our experimental model for evaluating allergic reactions in the respiratory tract suggested that the allergy-suppressing effect of the photocatalytic filter can be evaluated.

## 1. Introduction

In recent years, environmental policies have become an inevitable and important issue from the perspective of sustainable development, and various environmental policies are being implemented in various countries [1,2,3]. Air pollution caused by fine particulate matter (PM2.5), volatile organic compounds (VOCs), and bioaerosols has been recognized as an environmental risk with adverse health effects [4,5,6,7]. These air pollutants are also known to cause allergic diseases, such as asthma and allergic rhinitis, due to their involvement in airway inflammation, airway hyperresponsiveness symptoms, and allergic sensitization due to trans-airway exposure [6,8]. Although allergic disorders, such as asthma, are caused by sensitization due to inhalation of allergens, epigenetic mechanisms are known to play a crucial role in their pathogenesis through mediating the effects of environmental factors, including air pollutants [9,10]. Allergic symptoms are an economic concern as they interfere with daily life as well as occupational life, and affect productivity loss [11]. Besides medical treatment to suppress allergic reactions, reducing exposure as much as possible is important from the viewpoint of prevention.

It is known that photocatalytic reactions can decompose allergenic organic chemicals using reactive oxygen species produced by photocatalysis in the presence of titanium dioxide (TiO_2_) and light [12,13]. It is known that the photocatalytic efficacy of the filter is affected by the density and crystal structure of the TiO_2_ coating [14,15]. The development of more efficient photocatalytic filters has been progressing recently by controlling the combustion temperature of metals [14,16]. In particular, the low-temperature, high-velocity oxygen fuel (LT-HOVF) spraying method, which suppresses the combustion temperature, produces high-density TiO_2_ films with good adhesion to the substrate, and with minimal change to the crystal structure because the spray is applied to the substrate at a low temperature and high speed [16]. Photocatalytic filters produced by the LT-HOVF spraying method are expected to increase the efficiency of the photocatalyst and suppress allergic reactions.

There are some reports that evaluate the photocatalytic decomposition of allergenic substances [13,17,18,19], but these reports are mainly physicochemical evaluations, such as antigen reactivity and amino acid analysis of pollen and egg white albumin after photocatalytic treatment. To our knowledge, there are no reports to date that have investigated the actual effect of photocatalysts in suppressing allergic reactions. Furthermore, in the evaluation of devices, including photocatalysts for allergy decomposition, there are no reports of animal models being used to verify the suppression of allergic reactions.

Allergic reactions generally have a threshold at which an allergic reaction is triggered if an antigen enters the body [20,21]. In other words, even if exposure to allergens is reduced by photocatalytic decomposition, allergic reactions will be induced if there are antigens present that exceed the threshold. In order to evaluate the effectiveness of photocatalytic filters, it is, therefore, necessary to verify the suppression of actual allergic reactions. In this study, we used a mouse allergic model with intratracheal instillation of ovalbumin (OVA) after photocatalysis by a TiO_2_ filter produced by LT-HOVF spraying.

## 2. Materials and Methods

### 2.1. Animals

Male Balb/c mice (7 weeks old) were purchased from Japan SLC, Inc. (Shizuoka, Japan). The animals were kept in the Laboratory Animal Research Center of the University of Occupational and Environmental Health for 1 week with access to free feeding of commercial diet and water. All procedures and animal handling were performed according to the guidelines described in the Japanese Guide for the Care and Use of Laboratory Animals, as approved by the Animal Care and Use Committee, University of Occupational and Environmental Health, Japan (Approval Code: AE19-021. Approval Date: 20 December 2019).

### 2.2. OVA Sensitization

**Figure 1:**
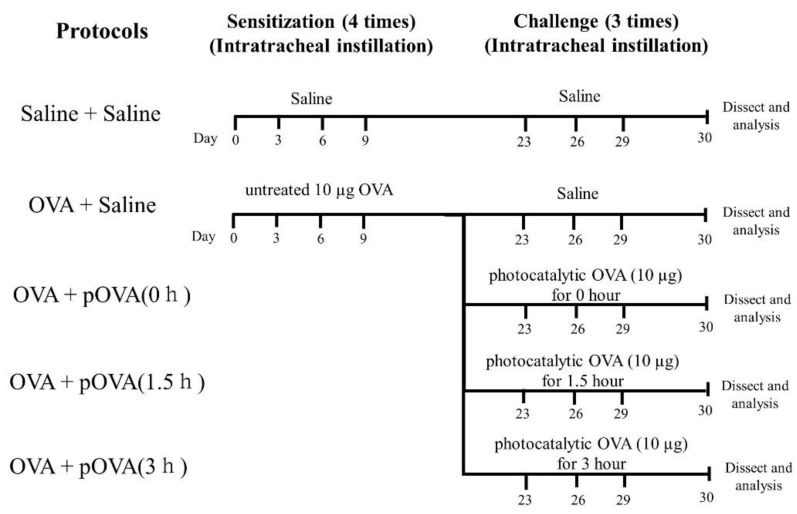
The protocol of the sensitization and the challenge of instillation used for mice. Photocatalytically untreated OVA was used for photocatalyst OVA for 0 h.

OVA (Kanto Chemical Co., Inc., Tokyo, Japan) was suspended in normal saline. A 10 µg dosage of OVA (200 μg/mL) was administered to mice (8 weeks old) via intratracheal instillations. The endotoxin concentration in the OVA solution prepared by the protocol was below 0.1 EU/mL, as measured by a ToxinSensor™ Chromogenic LAL Endotoxin Assay Kit (GenScript, Piscataway, NJ, USA). Mice were sensitized to OVA for 1, 4, 7 and 10 days, and intratracheally instilled with OVA on days 24, 27 and 30 (Figure 1). Non-sensitized animals were exposed to normal saline on the same exposure schedule.

### 2.3. OVA with Photocatalytic Treatment

**Figure 2:**
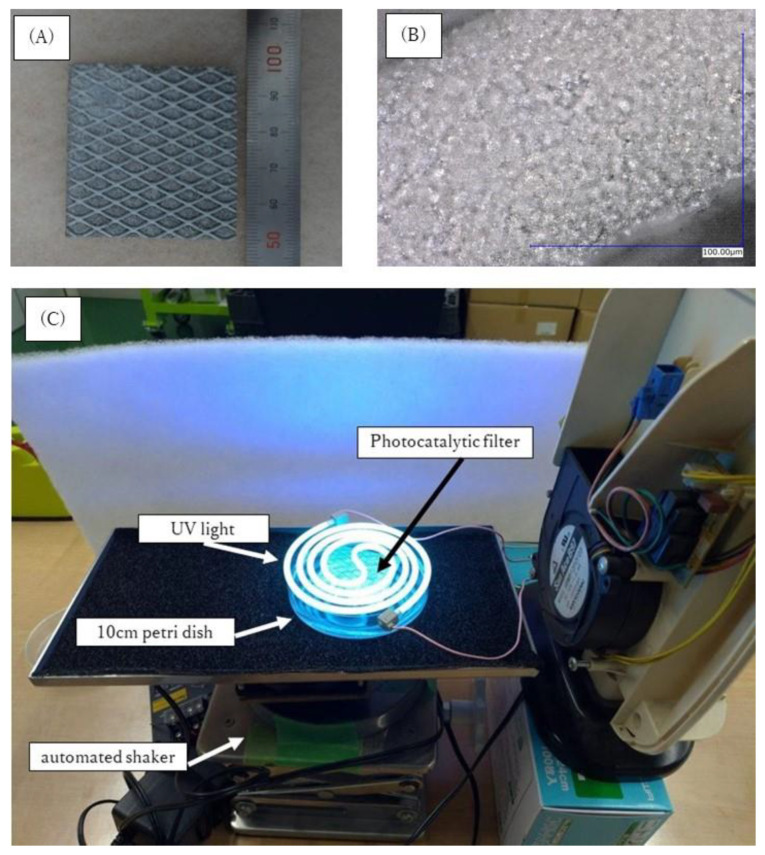
Equipment of the photocatalytic treatment for OVA. (**A**): the photocatalytic filter created by spraying titanium dioxide onto an aluminum fiber filter (size: 50 mm × 50 mm × 2 mm) using the LT-HOVF spraying method. (**B**): scanning electron microscope image of the photocatalytic filter. (**C**): layout of the equipment for photocatalytic treatment. A photocatalytic filter was placed in a 10 cm Petri dish and placed on an automated shaker while immersed in 30 mL of OVA solution (200 µg/mL) for 0, 1.5, and 3 h with UV irradiation.

The photocatalytic filter was provided by Fujico Co., Ltd., (Fukuoka, Japan). The filter was created by spraying titanium dioxide onto an aluminum fiber filter (size: 50 mm × 50 mm × 2 mm) using the LT-HOVF spraying method (Table 1). A thick layer of TiO_2_ was coated onto the aluminum fiber filter without any clogging between fibers, peeling, or missing film, as observed by scanning electron microscopy. The OVA solution with photocatalytic treatment was prepared by suspending OVA in normal saline. For photocatalytic treatment, a photocatalytic filter was placed in a 10 cm Petri dish and placed on an automated shaker while immersed in 30 mL of OVA solution (200 µg/mL) for 0, 1.5, and 3 h with UV irradiation (wavelength: 370 nm, intensity: 4.25 ± 1.17 mW/cm^2^) (Figure 2).

### 2.4. Instillation of OVA after Photocatalysis

OVA solutions after photocatalysis, prepared by the above protocols, were administered intratracheally to the mice at 50 μL/mouse. The endotoxin concentrations in the OVA solutions prepared by these protocols were all below 1 EU/mL, as determined by a ToxinSensor™ Chromogenic LAL Endotoxin Assay Kit (GenScript, Piscataway, NJ, USA). Photocatalytically untreated OVA was used for photocatalyst OVA for 0 h. A negative control group was intratracheally instilled with normal saline. The exposure groups (5 mice/group) for the present study were as follows: non-sensitized/saline challenged (sal-sal); OVA-sensitized/saline challenged (OVA-sal); OVA-sensitized/photocatalyst OVA challenged for 0 h (p-OVA (0 h)); OVA-sensitized/photocatalyst OVA challenged for 1.5 h (p-OVA (1.5 h)); OVA-sensitized/photocatalyst OVA challenged for 3 h (p-OVA (3 h)).

### 2.5. Analysis of Decomposition of OVA Photocatalysis

The concentrations of OVA (100 μg/mL) with photocatalytic treatment for 0, 1.5 and 3 h were measured using an ELISA kit (Prima Meat Packers, Ltd., Tokyo, Japan; Allergeneye^®^ ELISA II egg) according to the manufacturer’s instructions. The detection rates of OVA were calculated by setting the rate of photocatalysis for 0 h as 100%.

### 2.6. SDS-PAGE and Western Blotting

OVA solutions (1 mg/mL) with photocatalytic treatment for 0, 1.5 and 3 h were diluted to 500, 250, 125 and 62.5 μg/mL, respectively, and these OVA solutions were labeled with EzLabel FluoroNeo (ATTO Co., Tokyo, Japan; WSE-7010) for total protein detection and subjected to SDS-PAGE (15% polyacrylamide gel), followed by Western blot. Total proteins labeled with EzLabel FluoroNeo were irradiated with UV light and photographed after SDS-PAGE, according to the manufacturer’s instructions. OVA on the gel was transferred onto a PVDF membrane (Merck Millipore, Burlington, MA, USA) and blocked with 3% skimmed milk (MEGMILK SNOW BRAND Co., Tokyo, Japan) for 30 min. Then, 5000-fold-diluted HRP-conjugated ovalbumin polyclonal antibody (PA1-196-HRP, Thermo Fisher Scientific Inc., Waltham, MA, USA) was added and the resulting solution was incubated at room temperature for 1 h. The bound antibodies were visualized using an enhanced chemiluminescence kit (GE Healthcare, Chicago, IL, USA).

### 2.7. Animals following Intratracheal Instillation

The 5 mice in each group were anesthetized by inhalation of isoflurane (Pfizer Inc., New York, NY, USA) 24 h after the last instillation, and were then dissected. The mice in each group provided bronchoalveolar lavage fluid (BALF) for analysis. The lungs were inflated 3 times with 0.5 mL of saline by syringe, and BALF was collected from the entire lung in two or three portions. About 1–1.2 mL of BALF was collected by syringe. Serum samples were collected by cardiopuncture. Histopathological evaluation was performed with the left lung inflated and fixed with 10% formaldehyde after collecting BALF.

The obtained BALF was centrifuged at 800× *g* at 4 ℃ for 10 min, and the pellets were suspended in polymorphonuclear leukocyte (PMN) buffer (137.9 mM NaCl, 2.7 mM KCl, 8.2 mM Na_2_HPO_4_, 1.5 mM KH_2_PO_4_, 5.6 mM C_6_H_12_O_6_). The number of cells in BALF was counted using ADAM-MC (AR BROWN Co., Ltd., Tokyo, Japan), and the cells were splashed on a slide glass using cytospin. After the cells were fixed and stained with Diff-Quik (Sysmex Corp., Hyogo, Japan), the number of neutrophils, eosinophils and lymphocytes were counted by microscopic observation.

### 2.8. Analysis of Serum IgE and IgG1

Serum OVA-specific IgE and IgG1 antibodies were measured using Anti-Ovalbumin IgG1 (mouse) and Anti-Ovalbumin IgE (mouse) EIA Kit (Cayman Chemical Co., Ann Arbor, MI, USA). All measurements were performed according to the manufacturer’s instructions.

### 2.9. Histopathology

The obtained lung tissue, which was inflated and fixed with 10% formaldehyde under 25 cm water pressure, was embedded in paraffin, and 5 μm thick sections were cut from the lobe, then stained with hematoxylin and eosin.

### 2.10. Statistical Analysis

All results are expressed as the mean ± the standard error (SE) of mean. Statistical analysis was carried out using JMP^®^ Pro software (JMP Version 15, SAS Institute Inc., Cary, NC, USA). Analysis of variance and the Tukey-Kramer’s Honestly Significant Different Test were applied where appropriate to determine individual differences. *p* values < 0.05 were considered statistically significant.

## 3. Results

### 3.1. Analysis of the Decomposition Ability of OVA

**Figure 3:**
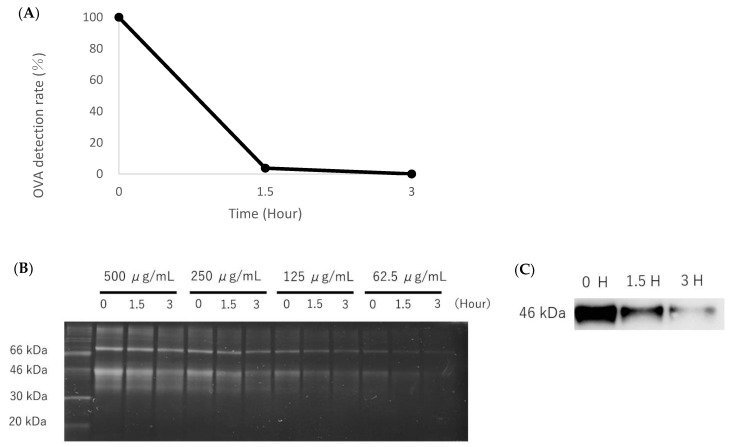
Analysis of the decomposition of OVA after photocatalysis. (**A**): the OVA detection rate of OVA solution (200 μg/mL) by photocatalytic treatment. (**B**): the detection of total protein in OVA solutions (500, 250, 125, 62.5 μg/mL) using SDS-PAGE. (**C**): the detection of OVA expressions by Western blotting (62.5 μg/mL). The detection of total protein in each OVA solution (500, 250, 125, 62.5 μg/mL) by photocatalytic treatment decreased in a time-dependent manner. The detection of OVA expressions in the solutions decreased in a photocatalytic time-dependent manner.

Figure 3 shows the results of the decomposition ability of OVA after photocatalysis. The detection rates of OVA with photocatalytic treatment were 3.8% and 0.096% for 1.5 h and 3 h, respectively (Figure 3A). The SDS-PAGE results showed that the protein expressions at 46 kDa decreased in a photocatalytic time-dependent manner (Figure 3B). The expression of OVA at 46 kDa also decreased in a photocatalytic time-dependent manner in the Western blot (Figure 3C).

### 3.2. OVA-Specific IgE and IgG Production

**Figure 4:**
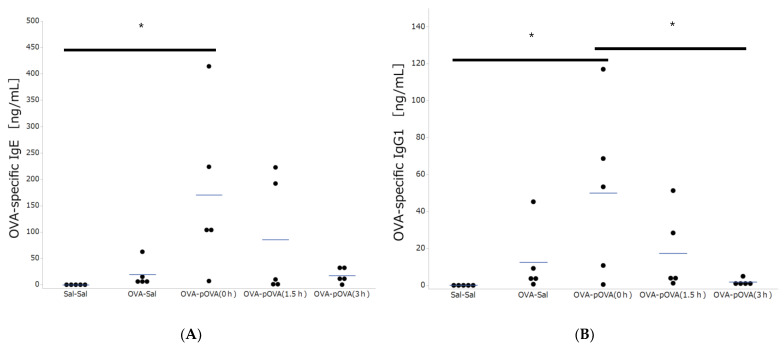
OVA-specific IgE and IgG production in serum. Blue bars represent the mean (*n* = 5/group). (**A**): OVA-specific IgE in serum. (**B**): OVA-specific IgG1 in serum. There was a significant increase in OVA-specific IgE and IgG in the p-OVA (0 h) group compared to the sal-sal group (negative control) and p-OVA (3 h) group. * *p* < 0.05 vs. other groups.

Figure 4 shows the results of OVA-specific IgE and IgG1 production in the serum of mice that were exposed to saline and OVA with photocatalytic treatment. Both OVA-specific IgE and OVA-specific IgG1 concentrations were under the detection limits in all the samples collected from the non-sensitized mice. Ovalbumin sensitization resulted in the production of antigen-specific IgE and IgG1. The levels of OVA-specific IgE were significantly higher than those for the non-sensitized mice (Figure 4A), and the levels of OVA-specific IgG1 were significantly higher than the sal-sal and p-OVA (3 h) treatment groups (Figure 4B). An upward trend of OVA-specific IgE and OVA-specific IgG1 concentrations, dependent on the level of photocatalytic degradation, was observed, and the level of increase in the p-OVA (3 h) treatment group was comparable to that in the OVA-sal treatment group.

### 3.3. Cell Analysis in BALF

**Figure 5:**
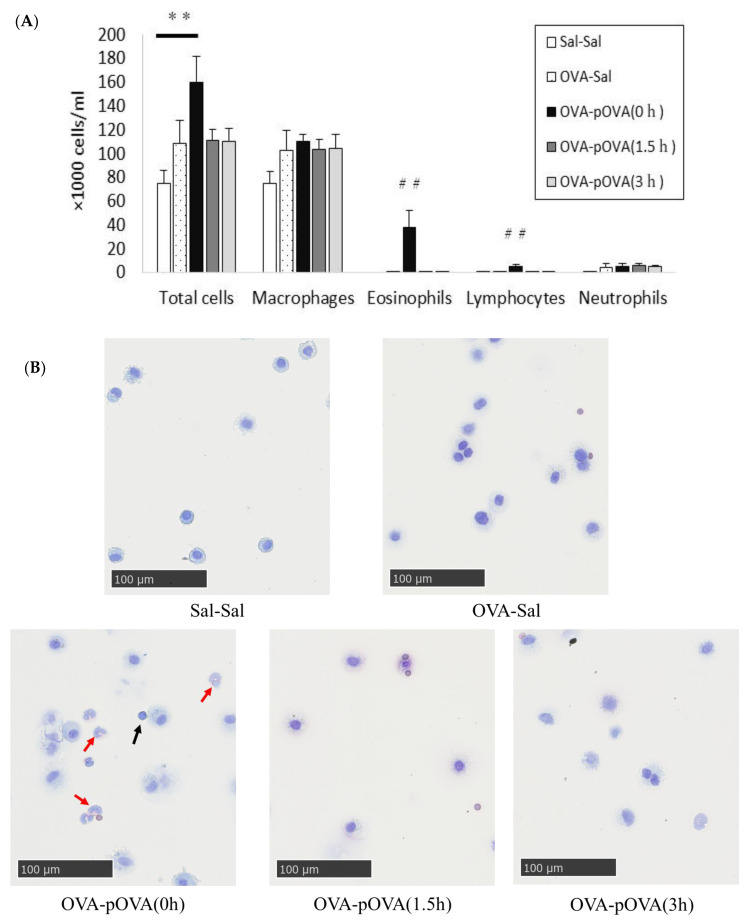
The inflammatory cells in BALF. (**A**): the distribution of inflammatory cells in BALF. Values are expressed as the mean ± SE (*n* = 4–5/group). ** *p* < 0.01 vs. sal-sal group (negative control). ^##^ *p* < 0.01 vs. other groups. (**B**): inflammatory cells in BALF with cytospin. The numbers of eosinophils and lymphocytes significantly increased in the p-OVA (0 h) group compared to other groups. Infiltration of eosinophils (red arrows) and lymphocytes (black arrows) in BALF with cytospin was observed in the p-OVA (0 h) group. Scale bar: 100 μm.

Figure 5 shows the cellular analysis of the BALF following the intratracheal instillation of OVA after photocatalysis. The numbers of eosinophils and lymphocytes were significantly higher in the p-OVA (0 h) treatment group than in the other groups. The total cell counts in the p-OVA (0 h) treatment group were significantly higher than in the sal-sal treatment group, used as a negative control, although there was not a significant increase in the neutrophil count in either group (Figure 5A). Infiltration of eosinophils was also observed in the BALF with cytospin in the p-OVA (0 h) treatment group (Figure 5B).

### 3.4. Pathological Features in the Mouse Lung

**Figure 6:**
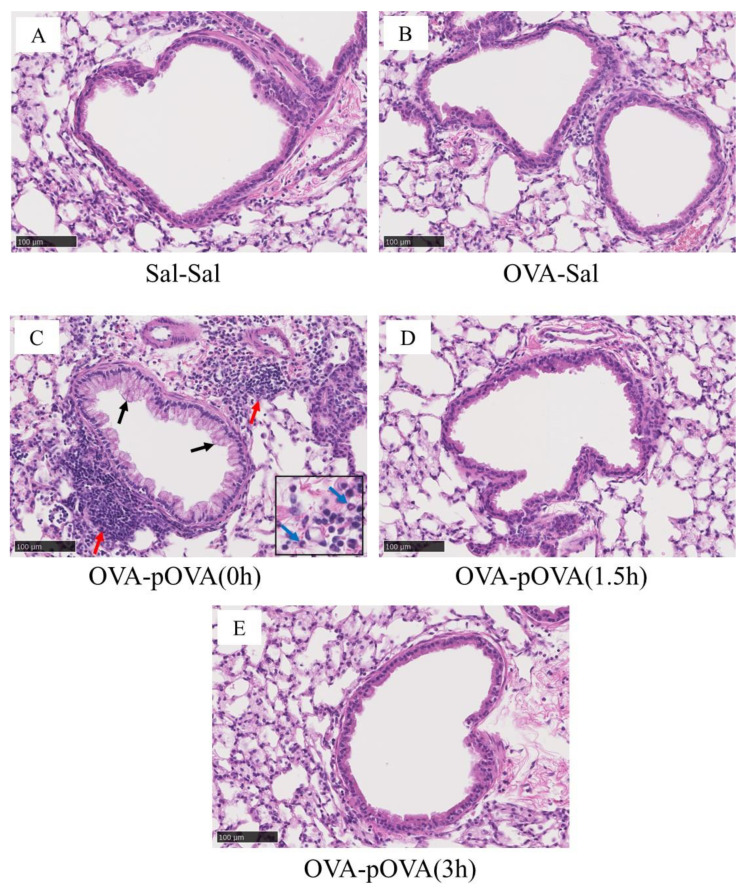
Histopathological findings of the lung after intratracheal instillation of testing samples (hematoxylin and eosin staining). (**A**): sal-sal group. (**B**): OVA-sal group. (**C**): p-OVA (0 h) group. (**D**): p-OVA (1.5 h) group. (**E**): p-OVA (3 h) group. In the p-OVA (0 h) treatment group, there was goblet cell hyperplasia (black arrows), infiltration of inflammatory cells around the bronchial wall (red arrows), and eosinophilic infiltration (blue arrows). In the p-OVA (3 h) and OVA-sal treatment groups, there were no changes in the bronchial epithelium or inflammatory cell infiltration; the levels were similar to that in the sal-sal treatment group used as a negative control. Scale bar: 100 μm.

Figure 6 shows the pathological findings in the mouse lungs. The lung histopathological findings for the p-OVA (0 h) treatment group included goblet cell hyperplasia, infiltration of inflammatory cells around the bronchial wall, and eosinophilic infiltration. In the p-OVA (1.5 h) treatment group, slight goblet cell hyperplasia and infiltration of inflammatory cells around the bronchial wall were observed, but this was very mild compared to the p-OVA (0 h) treatment group. In the p-OVA (3 h) and OVA-sal treatment groups, there were no changes in the bronchial epithelium or inflammatory cell infiltration; the levels were similar to that in the sal-sal treatment group, used as a negative control.

## 4. Discussion

In the present study, we conducted intratracheal instillation into mice using OVA after photocatalysis in the presence of a TiO_2_ filter produced by LT-HOVF spraying, in order to verify the suppression of the allergic reaction by the photocatalytic filter.

First, in order to validate the experimental model for evaluating the suppression of allergic reactions, such as asthma, we examined the protocol of sensitization. OVA has previously been used as an allergic substance in studies related to allergy [22,23,24,25]. Yu et al. tested inhalation exposure to PM2.5 and OVA as an animal asthma model [22]. There are many allergens in the indoor environment, such as mite antigens and house dust. Mite antigens have been reported to show an increase in IgE in serum similar to OVA [25]. Therefore, OVA is considered to induce allergic reactions similar to those induced by allergens in the indoor environment. In the present study, mice were sensitized four times and given intratracheal instillations of 10 µg/dose of OVA without adjuvant three times. In the p-OVA (0 h) treatment group, used as a positive control, we observed an allergic reaction in the form of an increase in the number of eosinophils in BALF, and OVA-specific antibodies in serum, 24 h after the final instillation; inflammatory cell infiltration around the bronchi and eosinophil migration were also observed in the lung histopathological findings. These observations suggested that an allergic reaction had been induced. Mlčková et al. reported sensitization by intratracheal instillation of 10 μg of OVA given three times, and found a significant increase in OVA-specific IgG and IgM [23]. Since allergic reactions have been induced even with exposure by intratracheal instillation of 0.5 μg/mouse [24], it is considered that the exposure dose used in our examination was sufficient to cause allergic reactions.

Next, we examined the inducibility of allergic reactions associated with asthma and the suppression of reactions according to the decomposition of OVA. The processes of allergic reactions associated with asthma generally involve airway inflammation, such as eosinophil infiltration, and the production of IgE antibodies, which occur within a few hours to 24 h after exposure, as well as airway epithelial goblet cell hyperplasia and airway wall thickening, which occur within a few days after repeated exposure [26]. Since the same pathology was observed in the present study, the protocol of the present study is considered to be representative of the pathology of asthma. In the present study, the intratracheal instillation of OVA after photocatalysis resulted in no increase in the number of eosinophils in BALF, and in a photocatalytic time-dependent decrease in IgG1 and IgE. Because we did not evaluate lung function, it was unclear whether the suppression of the increase in the number of eosinophils in the BALF reflected a suppression of asthmatic response. However, eosinophil concentrations in sputum are used as one of the biomarkers of asthma severity in humans [27]. Half of patients with more than 3% of eosinophils in the sputum have been reported to have severe asthma [28]. Considering that the increase in the number of eosinophils in BALF may also reflect an allergic reaction, the photocatalytic decomposition of OVA may have suppressed the allergic reaction.

In general, allergic reactions can be triggered when even a very small amount of allergen gets into the body if the living body is sensitized to the allergen. On the other hand, there is a threshold level at which allergic reactions can occur [20,21]. In desensitization therapy for egg allergies, it has been reported that ingestion is started below the threshold level, and so does not produce allergic symptoms [20]. It has been reported that there is a threshold level of inhalation exposure (30 min/week, 6 weeks) to OVA of 0.1–3.3 mg/m^3^ that does not induce anti-OVA IgE and IgG in serum in rats [21]. It is considered that when allergens enter the body, they bind to multiple IgE antibodies on mast cells and basophils, then the mast cells and basophils degranulate, and inflammatory mediators are released; however, plasma cells continue to proliferate and produce excessive amounts of IgE or IgG [29,30]. From the viewpoint of the mechanisms of immune response, the decrease in IgE and IgG1 that we observed may have reflected suppression of the asthmatic response. The OVA became inactive after photocatalytic treatment for 1.5 to 3 h, which may have suppressed further antibody production and subsequent allergic reactions.

In the normal HOVF method, TiO_2_ is sprayed at a high speed onto a base material, and it has been reported to be highly durable and adhesive to the base material. On the other hand, in order to increase the photocatalytic efficiency of the filter, it is necessary to control the pores caused by oxidation of the sprayed material and the changes in the crystal structure caused by high-temperature irradiation [16]. In the LT-HOVF method, raw TiO_2_ powder is sprayed onto the substrate at a low temperature and high speed, with little effect of denaturation by mixing with the high-pressure atmosphere; thus, the crystal structure of the TiO_2_ is retained in a fine state, and the porosity of the TiO_2_ coating is decreased and good photocatalytic efficiency can be obtained [31]. Regarding the decomposition of OVA after photocatalysis, the detection rate (D) of the OVA content when using TiO_2_ powder has been shown to be 26.8% at 0.5 h of photocatalysis compared to before photocatalysis, and a decrease in all amino acids after photocatalysis has been observed [18]. In the present study, the detection rate of OVA was 3.8% at 1.5 h of photocatalysis, and assuming that the photocatalysis in the present study had been performed for 0.5 h, the level of protein degradation would have been similar (D = $\sqrt[{3}]{0.038}$ × 100 = 33.6%). The TiO_2_ filter produced by LT-HOVF spraying was considered to have a photocatalytic efficiency comparable to that of raw TiO_2_ powder.

In general, the mechanism of photocatalytic decomposition is the generation of negative electrons (e^−^: electron holes) and positive holes (h^+^) by exposure to UV light and TiO_2_. The generated e- reacts with oxygen (O_2_) in the air to produce superoxide anions (O_2_^−^) (O_2_ + e^−^→O_2_^−^). Next, h + reacts with H_2_O to produce hydroxyl radicals (.OH) (OH^−^ + h^+^→.OH). The hydroxyl radicals (.OH) resulting from these processes have a much higher oxidizing power than chlorine or ozone, and are useful for the oxidative decomposition of organic compounds [32]. Although the photocatalytic decomposition of organic substances has not been fully elucidated, it is thought that the OH radicals generated by UV light and TiO_2_ act on proteins and peptides to deprive them of electrons, resulting in oxidative cleavage and decomposition [18,33,34]. Considering that the detection of protein decreased in a photocatalytic time-dependent manner in the SDS-PAGE, and the labeling of polyclonal OVA-specific antibodies also decreased, it is possible that some of the compositional proteins of OVA were degraded to at least the amino acid level, and the functional groups labeled by the OVA-specific antibodies were also decomposed.

## 5. Conclusions

In the present study, in order to verify the suppression of the allergic reaction by the photocatalytic filter, we instilled OVA, after photocatalysis, into the trachea of mice and evaluated the allergic reactions. Our experimental system was designed to be an allergic asthma model, and allergic reactions resulting from inflammation of the airways and production of OVA-specific antibodies were induced. In fact, using this model, we verified the effectiveness of the photocatalytic filter produced by the LT-HOVF method in suppressing allergic reactions, and the decomposition of OVA after photocatalysis using the photocatalytic filter was found to actually suppress allergic reactions in mice. Taken together, our experimental system for evaluating allergic reactions in the respiratory tract suggested that the allergy-suppressing effect of the photocatalytic filter can be evaluated.

## Figures and Tables

**Table 1:** Physicochemical characterization of TiO_2_.

Physicochemical Properties	Value
Primary size	21 nm
Specific surface area (BET)	50 ± 15 m^2^/g
Crystal structure (Anatase:Rutile)	4:1
Thickness of TiO_2_ layer sprayed on the aluminum fiber	2 μm

## Data Availability

The data that support the findings of this study are available from the corresponding author upon reasonable request.
